# Performance of Nano-Submicron-Stripe Pd Thin-Film Temperature Sensors

**DOI:** 10.1186/s11671-016-1565-8

**Published:** 2016-07-28

**Authors:** Xiaoye Huo, Jingjing Xu, Zhenhai Wang, Fan Yang, Shengyong Xu

**Affiliations:** Key Laboratory for Physics and Chemistry of Nanodevices and Department of Electronics, Peking University, Beijing, 100871 People’s Republic of China

**Keywords:** Built-in temperature sensor, Submicron thermometry, Nanostripe, Sensitivity, Thin-film

## Abstract

Dozens of small dual-beam thin-film temperature sensors with a total width down to 430 nm were fabricated and tested. The sensors were all made from 90-nm-thick Pd thin films, where the width of the narrow stripes was 70–100 nm and that of the wide ones was 210–800 nm. Two different calibration methods showed consistent and repeatable sensitivities of 0.7–1.2 μV/K for the sensors, confirming that the sensitivity mainly depended on the width configuration of each sensor. By integrating arrays of such sensors on a practical testing platform using hybrid e-beam lithography and photolithography techniques, we demonstrated that these sensors were capable of detecting a weak surface temperature difference of 0.1–0.2 K at microscale, and they could be scaled up as built-in temperature sensors in many practical devices.

**Electronic supplementary material:**

The online version of this article (doi:10.1186/s11671-016-1565-8) contains supplementary material, which is available to authorized users.

## Background

The rapid development in micro/nanoscience and technology and the emerging trend towards *Internet of Things* (IoT) [1] have aroused a tremendous demand for a variety of novel sensors with small size, high sensitivity, and accuracy, as well as high stability and reliability. Among them, temperature sensors at micro-/nanoscale are extensively demanded. Temperature measurement techniques are usually classified into two categories depending on their working modes: non-contact techniques and contact techniques. Over the past two decades, a number of non-contact techniques have been reported for temperature sensing at small scales. These techniques employ micro-/nanosized fluorescent indicators for temperature detection, such as quantum dots [2, 3], organic dyes [4, 5], metal-organic frameworks [6, 7], fluorescent proteins [8, 9], nanoparticles [10], and nanodiamonds [11, 12]. They have the merits of high spatial resolution to nanoscale, quick response time as small as microseconds, and are more suitable for two-dimensional (2D) mapping of local temperatures. However, besides a low temperature resolution, a non-contact technique requires a complicated optical system and exposure of the subject under test to the probing beam. These requirements make them not suitable for applications of most flexible electronics and wearable systems, which are hot issues in fields of fundamental research and industry. Contact sensors are more applicable for wearable electronics and systems, especially built-in sensors made from 2D materials [13]. Among the several currently available built-in thermal sensors, conventional thin film thermocouples have advantages in high spatial resolution around 1 μm [14], a high temperature resolution better than 10 mK [15], and fast response time of 1-10 μs [16, 17].

Recently, we developed a novel type of thermal sensor, which had a dual-beam structure and was made from a single layer of metallic thin film [18–20]. By using these sensors with a dual-beam configuration of 30–3 μm, 2D mapping of temperature difference at the 40–100 K level and over an area of 0.7 mm × 0.7 mm was demonstrated [19]. The same working mechanism was found valid in smaller sensors, and the total size of the senor was made down to 900 nm [20]. The submicron sensors have a promising potential for application in novel microelectrical devices [21] and in microfluidic devices for local chemistry and bio-sensing [22–25]. For example, polymerase chain reaction (PCR) process relies on thermal cycling, i.e., repeated heating and cooling for DNA melting and enzymatic replication, so the thermal detection and control during the process is crucial [25].

Intracellular temperature sensing has become a hot topic recently. A real-time mapping of the subcellular temperature may greatly help the study of gene expression, enzyme reaction, and other cellular activities [26, 27]. For instance, Okabe et al. found that the temperature of nucleus and centrosome of many cells was higher than that of cytoplasm by 0.7–0.9 K in different cycle stages [28], indicating that a lot more was unknown in cell biology at the sub-cell level. With more and more doubts rising about the intracellular temperature measured with the non-contact techniques [29], contact sensors with high temperature sensitivity and high spatial resolution which can verify the intracellular temperature are in great demand. Though solid temperature sensors (working with contact mode) such as thermocouples usually show a high temperature sensitivity, they often have a large size much more than 1 μm and are therefore not considered as the first choice for temperature sensing at the submicron scale. For application at the submicron and nanoscales, solid temperature sensors should be made into smaller size meanwhile kept the high sensitivity. Recently, Wang et al. fabricated a novel tip-shaped thermocouple and successfully detected a weak change in intracellular temperatures of individual cells [30].

In this work, we report the results on the fabrication and performance of dozens of Pd nano-submicron-stripe thin-film temperature sensors. By using a better fabrication procedure, we have squeezed the total width of the smallest dual-beam sensors down to 430 nm, less than half of the previous record [20]. Calibrated with two different methods, these sensors showed repeated sensitivity, which depended mainly on the width configuration of the two stripes and was consistent with previous results [20]. We also demonstrated that these sensors could be made into practical testing devices as built-in sensors by using a combined fabrication technique, where the submicron sensors made with e-beam lithography could be overlapped on large lead patterns made with photolithography technique. In such a hybrid device, these sensors remained a stable and repeatable sensitivity for sensing a weak surface temperature difference of 0.1–0.2 K over a local area of 10–100-μm scale.

## Methods

The sensors were made with pure Pd thin films, which were deposited with an electron-beam evaporator (AXXIS, Kurt J. Lesker) on 4-in SiO_2_/Si wafers. The 2-μm-thick SiO_2_ served as an insulating layer on the 500-μm-thick Si (100) wafer. For the fabrication of nano-submicron-stripe dual-beam sensors, the device patterns were created with standard electron-beam lithography (EBL) processes on a Raith 150-II system at an operation voltage of 20 kV, an aperture dimension of 20 μm, a step size of 8 nm, and a writing field of 100 μm. EBL resist PMMA solution (Mw = 950 K, A4, Allresist) was spin-coated on the wafer with a programmable spin coater (Solarsemi Easyline 200 TT) at 4000 rpm for 1 min and baked at 170 °C for 3 min. Then, a 90-nm-thick Pd thin film was deposited with e-beam deposition at a speed of 0.2 nm/s. Before that, a 0.5-nm-thick Cr layer was deposited as the adhesive layer. Final device pattern was obtained after standard lift-off process in pure acetone.

To make onsite Cr/Pd control sensors for calibration, a 50-nm-thick Cr layer was deposited with the e-beam evaporator, and the 90-nm Pd layer was patterned and deposited together with the dual-beam Pd sensors. The widths for the Cr and Pd stripes were made the same, and dozens of Cr/Pd control sensors with six different widths were fabricated on each single device. For fabrication of the hybrid testing platform, patterns of the outer leads, onsite heater, and big contact pads (shown in Fig. 4) were all fabricated with standard photolithography processes with AR-P5350 photoresist (Allresist). An oxygen plasma was applied to remove the residual photoresist before deposition of a 90-nm-thick Pd thin film.

The thickness of the Pd thin films was determined with an atomic force microscope (AFM, Veeco D3100). Micrographs of the samples were all taken with a field emission-environment scanning electron microscope (SEM, FEI_XL30 SFEG) operating at an accelerating voltage of 20 kV. Electrical measurements were performed on a standard electrical probe station (EB-6). The direct current was applied with a source meter (Keithley 2400), and the output voltage of each sensor was measured with a nanovoltmeter (Keithley 2182A), both controlled with a homemade computerized data acquisition system using Labview program. The output voltage for each sensor usually stabilized after 10–40 s, depending on its distance to the heating zone, and the average stabilized voltages corresponding to different heating powers were used as the experimental values.

## Results and discussion

**Fig. 1:**
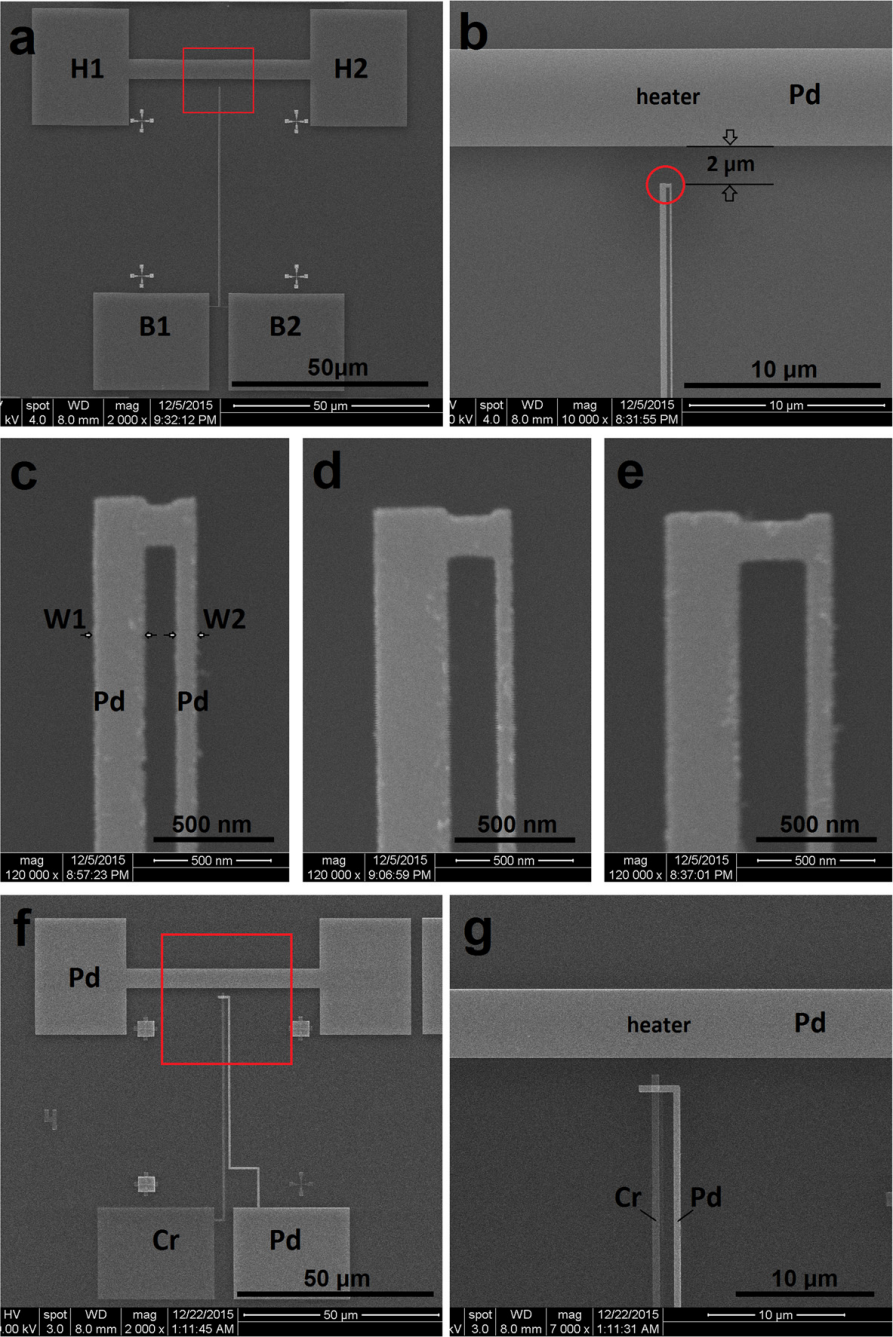
SEM micrographs of the nano-submicron-stripe dual-beam Pd temperature sensors and an onsite Cr/Pd control sensor. **a** A device with an onsite stripe heater and a temperature sensor. **b** An enlarged image of the sensing area highlighted with a red frame in **a**. The distance from the heater to the junction is 2 μm. **c–e** Magnified SEM micrographs of the sensing region of Pd sensors as highlighted in **b**.These sensors have total widths of 430, 590, and 710 nm, with stripe widths of 210–80, 310–70, and 310–100 nm, respectively. **f** SEM micrograph of onsite Cr/Pd control sensors with same shape and material of the onsite heater as Pd sensors in **a. g** Magnified SEM micrograph of the sensing area as highlighted in **f**. The beam width is 500 nm

Figure 1 shows SEM micrographs of several typical nano-submicron-stripe dual-beam Pd sensors and an onsite Cr/Pd thin film thermocouple. Dozens of the Pd sensors are patterned into a dual-beam structure with the narrow beam of 70–100 nm in width and the wide ones of 210–800 nm with EBL and e-beam evaporating techniques. As shown in Fig. 1a,b (which is an enlarged image of the area highlighted with a red frame in Fig. 1a), an onsite straight-bar heater (45 μm-long, 5 μm-wide) also made of the same Pd thin film as the sensors is located 2 μm away from the junction part (hot end) of the sensor. In Fig. 1a, the two contact pads of the heater are marked with “H1” and “H2,” and the contact pads (cold ends) of the sensor are marked with “B1” and “B2”. When applying a DC current from 0 up to 50 mA to the heater, the heating power is adjustable from 0 to over 100 mW. The resistance of the heater is measured to be 41.7 ± 0.1 Ω under a small excitation current. The onsite heater thus generates a local temperature difference between the hot end and cold end of the sensor, leading to a DC voltage output between the two cold ends of B1 and B2.

In this work, dual-beam Pd sensors are made into five different width configurations of W1–W2, where W1 and W2 refer to the widths of the wide and narrow beams, as shown in Fig. 1c. These configurations are 310–100, 310–80, 310–70, 210–80, and 210–70 nm, respectively. Figure 1c–e shows SEM micrographs for the sensing region of individual Pd sensors with the configuration of 210–70, 310–70, and 310–100 nm, respectively. The total width of the 210–70 nm sensor is 430 nm (including the spacing between the two beams).

The onsite Cr/Pd sensor shown in Fig. 1f is fabricated for in situ calibration of the Pd dual-beam sensors. Figure 1g is the magnified image of the marked area with a red frame in Fig. 1f. The shape and material of the onsite heater, as well as the spacing between the heater and the Cr/Pd sensor junction are made exactly the same as those shown in Fig. 1a. In order to obtain reliable calibration results, each beam of the control samples was made into several different widths. The one shown in Fig. 1f, g has a beam width of 500 nm.

**Fig. 2:**
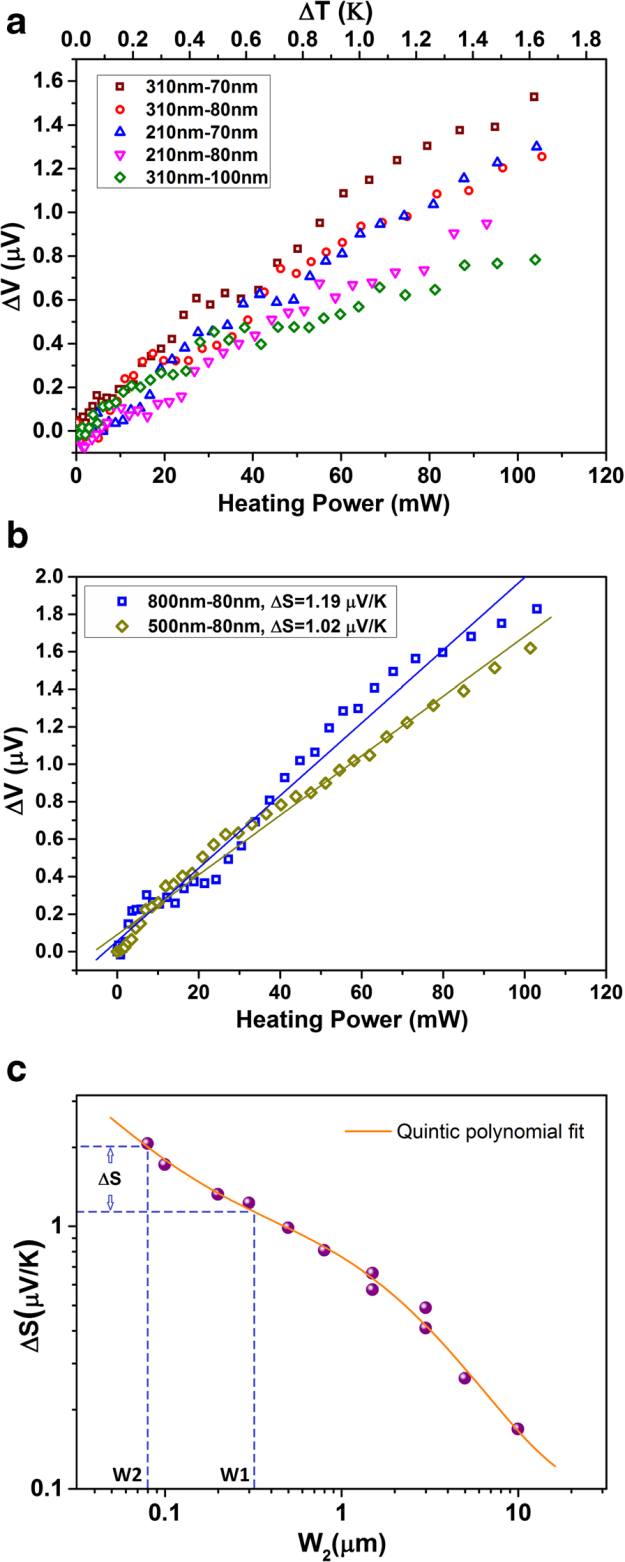
Measurement results of dual-beam Pd sensors and calibration results with on-site Pd thermocouples. **a** Voltage response under different heating powers of five different single-layered temperature sensors. The top *x*-axis shows the calibrated temperature difference. **b** Measurement results of two Pd sensors with width configurations of 800–80 and 500–80 nm. The Δ*S* values of the two sensors are obtained from our previous work [20]. **c** Δ*S* values of the sensors with different narrow beams from our previous work [20]. The wide beam is fixed at 100 μm in width, and the width of the narrow beam varies from 80 nm to 10 μm. The data are plotted in log-log scale. The *orange line* shows the quintic polynomial fitting of the data. The *dashed blue lines* indicate the method of calculating Δ*S* for a Pd sensor with a width configuration of 310 and 80 nm

The performance of nano-submicron-stripe dual-beam Pd sensors is presented typically in Fig. 2a. For the sensors with five different beam width configurations, namely 210–70, 210–80, 310–70, 310–80, and 310–100 nm, the 310–70-nm sensor has the highest sensitivity. The data for output voltage versus heating power of the onsite heater are nearly linear.

Figure 2b shows the measurement results of two Pd sensors with width configurations of 800–80 and 500–80 nm. The data are linearly fitted. The sensitivity (defined as Δ*S* = Δ*V*/Δ*T*) values of the two sensors, Δ*S* = *S*1 − *S*2, where *S*1 and *S*2 are the Seebeck coefficients for the wide and narrow beams, have been calculated to be 1.19 and 1.02 μV/K, respectively, in our previous work [20]. Since the materials and film thickness used in this work are the same as our previous work, and Seebeck coefficient is an intrinsic value that is determined by the density of conduction electrons and the scattering rate [31], we assume here that the two sensors shown in Fig. 2b have the same sensitivities with previous data. With this method, we obtain the corresponding temperature difference (Δ*T*) between the sensor junction region and the cold ends versus the heating power of the onsite heater. These data are shown with the top *x*-axis in Fig. 2a.

By simple linear fittings, the sensitivities of the five different kinds of sensors are derived, shown in Table 1. A comparison is made with the data of our previous work [18] shown in Fig. 2c. As the Pd sensors in the previous work [18] have thickness of 100 nm and the sensors in this work have thickness of 90 nm, the comparison in Fig. 2c has an error of about 10 %. Here, the measured data of Δ*S* are the differences between the Seebeck coefficients of under-test Pd stripes and a 100-μm-wide Pd stripe [18, 20] and taken from the average of the two calibration methods mentioned below. More accurate Δ*S* values are calculated by using a quintic polynomial fitting. We calculate the expected Δ*S* values from the fitting curve and compare them to the measured data. In Fig. 2c, the dashed blue lines illustrate how the calculated Δ*S* is obtained for a dual-beam Pd sensor with a width configuration of 310–80 nm. The results are listed in Table 1. It shows that the measured data match remarkably well to the calculated data, indicating a high repeatability of the sensors. The uncertainty of the calibrated Δ*S* is estimated to be 5–8 %. For smallest sensors of 210–70 and 210–80 nm combinations, their sensitivities are measured to be 0.84 ± 0.04 and 0.69 ± 0.04 μV/K, respectively.

**Table 1:** Comparison between the calculated Δ*S* values and measured Δ*S* values of submicron Pd temperature sensors

Width W1–W2 (nm)	310–70	210–70	310–80	210–80	310–100	510–80	810–80
Calculated Δ*S* (μV/K)	0.93	0.83	0.84	0.75	0.49	1.08	1.26
Measured Δ*S* (μV/K)	0.96 ± 0.05	0.84 ± 0.04	0.82 ± 0.04	0.69 ± 0.04	0.50 ± 0.03	1.00 ± 0.05	1.22 ± 0.06

**Fig. 3:**
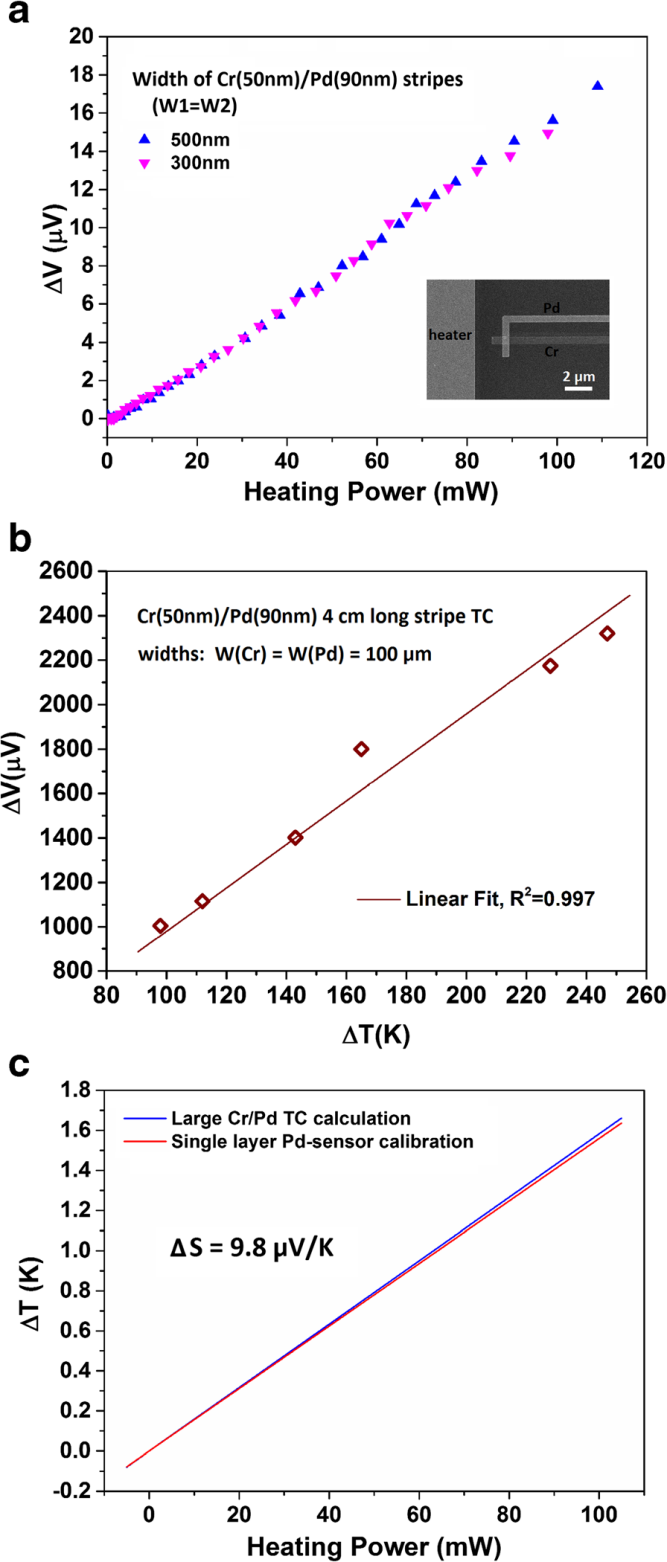
Calibration results with Cr/Pd thin film thermocouples. **a** Calibration result of Cr/Pd onsite thermocouples with beam widths of 500 and 300 nm. The inset is SEM micrograph of sensing region of the Cr/Pd sensor with a local heater. **b** Measured data of 4-cm-long Cr/Pd thin film thermocouples with stripe width of 100 μm. The temperature is calibrated with a homemade platform [31]. The data are linearly fitted. **c** Temperature increases under different heating powers of 4-cm-long Cr/Pd thermocouple and single-layer Pd sensors. The calibration results of the two methods match well with each other. The Δ*S* value is calculated as 9.8 ± 0.5 μV/K based on **a** and **b**

We also conducted calibration with onsite control samples made into conventional thermocouple structure with Cr (50 nm) and Pd (90 nm) thin film stripes, as typically shown in Fig. 1f, g. The inset of Fig. 3a shows the sensing region of the Cr/Pd sensor. The measurement results for control samples with a beam width of 300 and 500 nm are plotted in Fig. 3a. They share a similar slope for output voltage over heating power. Assuming each individual sample senses the same temperature difference Δ*T* under the same heating power [20], the data in Fig. 3a indicates that they have the same sensitivity Δ*S*_Cr-Pd_. Here, Δ*T* refers to the temperature difference measured at the sensing region under certain heating power and usually it takes around 20 s for temperature to stabilize and the whole measurement process for each data point takes 1–2 min.

To determine the absolute value of this Δ*S*_Cr-Pd_, we have fabricated 4-cm-long 100-μm-wide Cr/Pd thin film thermocouples on 2-in glass wafers and obtained their sensitivity by measuring the output voltage under a large temperature difference between the hot and cold ends of these sensors [32]. The measured data are plotted in Fig. 3b. With a linear fitting of the data, we get the sensitivity Δ*S*_L_ of these long Cr/Pd thin film sensors to be 9.8 ± 0.5 μV/K. We assume that Δ*S*_Cr-Pd_ is the same as that for the long Cr/Pd thin film thermocouples, i.e., Δ*S*_Cr-Pd_ = 9.8 ± 0.5 μV/K. Considering the size effect of Seebeck coefficient observed previously [18, 20], the real value of the onsite small Cr/Pd sensors, Δ*S*_Cr-Pd_, could be slightly smaller.

With the sensitivity Δ*S* of 9.8 ± 0.5 μV/K, the corresponding temperature difference Δ*T* of each heating power is determined by Δ*T* = Δ*V*/Δ*S*. The results are plotted in Fig. 3c. We also obtain the curve of ΔT versus heating power for the single-layer Pd sensors from Fig. 2b in the same way. Surprisingly, the curve of Δ*T* versus heating power calibrated with 4-cm-long Cr/Pd thermocouples matches well with the results of the dual-beam Pd sensors of 800–80 and 500–80 nm, as shown in Fig. 3c. This suggests that our dual-beam Pd sensors have the same reliability as the conventional Cr/Pd thin film thermocouples.

The performance of our nano-submicron-stripe dual-beam Pd sensors is further verified on a testing platform. To avoid temperature increase at the cold ends of the sensors while applying heating power at the sensing region, ideally, the cold ends should be far away from the hot zone. Also, on one measurement chip, a sensor array should be fabricated to allow multiple measuring spots. These two requirements lead to a large size of the testing platform. To reduce the cost and fabrication complexity, we have used a hybrid process that combines electron-beam lithography and photolithography techniques for patterning the wafer-size platform with nano-submicron sensors.

**Fig. 4:**
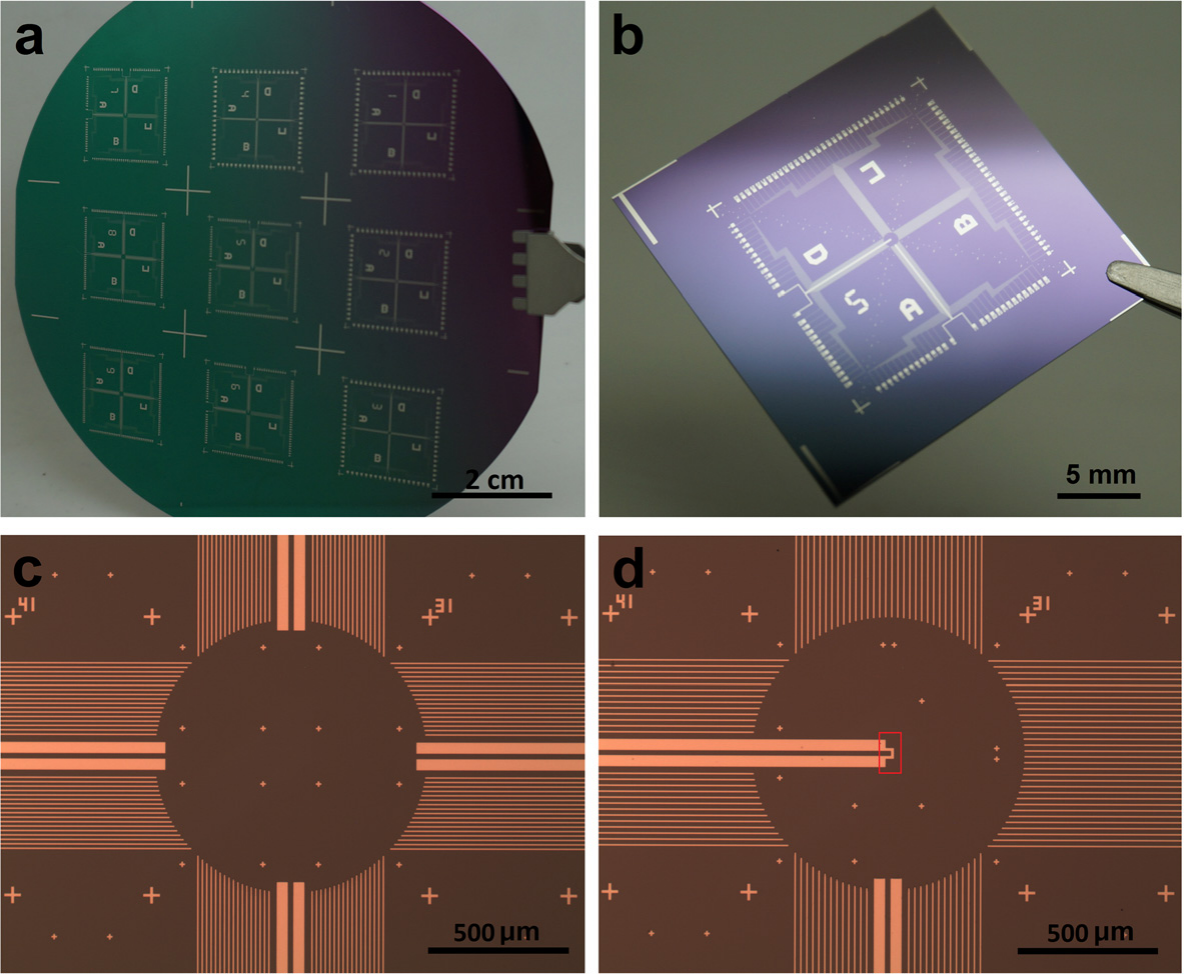
Photographs of the measuring system fabricated with photolithography. **a** Photograph of the 4′ silicon wafer with nine devices in a 3 × 3 matrix. **b** Photograph of one device with a size of 2.5 × 2.5 cm^2^. It contains 72 to 128 contact pads. **c** Microscopy picture of one central circular sensing region without local heater. The central circle is 1000 μm in diameter. The narrow lines are 5 μm in width, and the wide lines are 30 μm for heaters. **d** Microscopy picture of one central sensing region with an onsite heater highlighted with red frame

Figure 4 presents photographs of the platform fabricated with standard lithography. The patterns are made with a 90-nm-thick Pd thin film. On each 4-in SiO_2_/Si(100) wafer, nine devices in a 3 × 3 matrix are fabricated (Fig. 4a). On each single device, which is 2.5 × 2.5 cm^2^ in size as shown in Fig. 4b, each side of the four contains 18–32 contact pads. Altogether, 72 to 128 electrode leads, with width of 5 μm, connect these pads to the central region of the testing area, leaving open ends in a circular shape of 1000 μm in diameter, as shown in Fig. 4c. This empty space is left for the following e-beam lithography process. Cross markers with different size are pre-patterned with the contact leads, all made from a single layer of Pd thin film. The 30-μm-wide beams are prepared for connecting local heaters at the central region. For some devices, an onsite heater is pre-patterned in the center of the testing region, as highlighted with a red rectangle in Fig. 4d. The heater can generate a local heating power of hundreds of mW, and it plays an important role in the testing procedure.

**Fig. 5:**
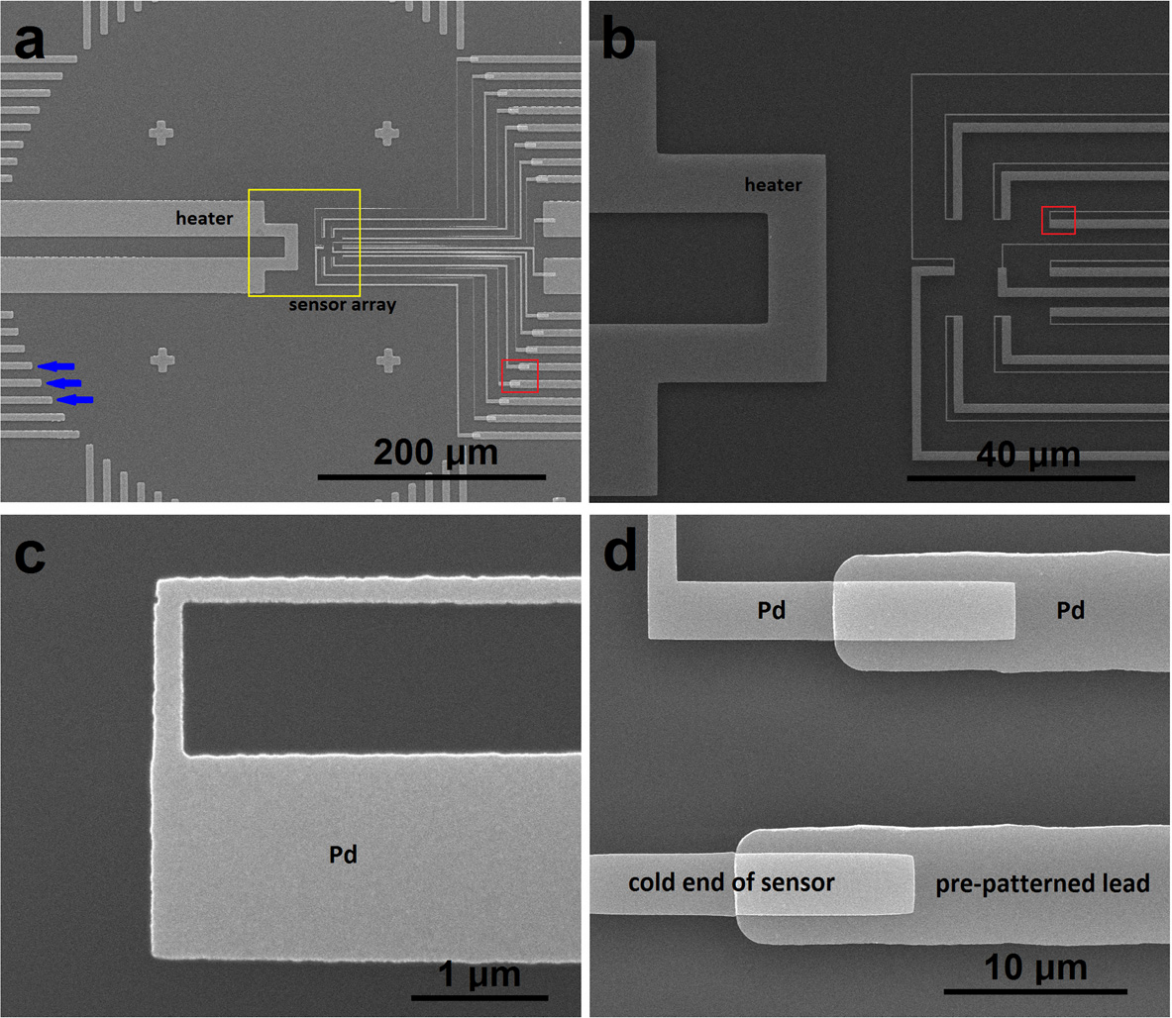
SEM micrographs of the central region of the platform. **a** SEM picture of a sensor array of nine dual-beam sensors and an onsite heater. *Three blue arrows* point the pre-patterned Pd leads. The beams of the sensors are 200-μm-long approximately. **b** Magnified SEM micrograph of the central sensing part marked with a *yellow frame* in **a**. The heater stripe is 5 μm in width, and the sensor array contains nine sensors in a 3 × 3 array in a 15 × 15 μm^2^ area. **c** Enlarged SEM image of the sensing part marked with a *red frame* in **b**. The configuration of the sensor is 1.5 μm–200 nm in width. **d** Overlapping region of the lithographic leads and EBL beams marked with a *red frame* in **a**. The wide beams are pre-patterned photolithographic leads, and the narrow beams are EBL-made cold ends of the sensors. They are all made of 90-nm-thick Pd thin film with overlapping length of 10 μm

On the as-fabricated device chip with pre-patterned Pd thin film for contact pads, long leads, local markers, and onsite heater, EBL and thin film deposition processes are employed for fabrication of the dual-beam Pd sensors. These sensors are made in the empty central region of each device, and the cold ends of the Pd sensors are overlapped with the pre-patterned Pd leads, as shown in Fig. 5. Figure 5 presents typical SEM micrographs for the central region of each platform. Figure 5a shows an overall view of a sensor array of nine dual-beam sensors, which is fabricated on the right side of an onsite heater with a distance of 20–40 μm. Three blue arrows at the bottom left point spare open ends of the pre-patterned Pd leads. While on the right side, 18 cold ends of the nine sensors in a 3 × 3 matrix are connected to the pre-patterned leads. This is a small array. Our multiplexer developed recently is capable to measure an array of up to 100 individual sensors. Figure 5b gives a close look at the sensor array for the region marked with a yellow frame in Fig. 5a, which is arranged in a 15 × 15 μm^2^ area with a spacing of 7.5 μm between two adjacent sensors. The image of the sensor marked with a red frame in Fig. 5b is further enlarged and shown in Fig. 5c. It has a dual-beam width configuration of 1500–200 nm. Figure 5d is a magnified image of the contact region where the cold ends of the sensors overlap the pre-patterned leads, as marked with a red frame in Fig. 5a. For enough misplacement tolerance, the overlapping area is 10 μm in length. With the position information provided by the cross markers, the sensor array fabricated with EBL can be overlapped precisely on the pre-patterned leads. The Δ*S* value of the sensor is calibrated to be 0.75 μV/K. This sensitivity is similar to those for sensors of 210–70 and 210–80 nm combinations, which are 0.84 ± 0.04 and 0.69 ± 0.04 μV/K, respectively.

**Fig. 6:**
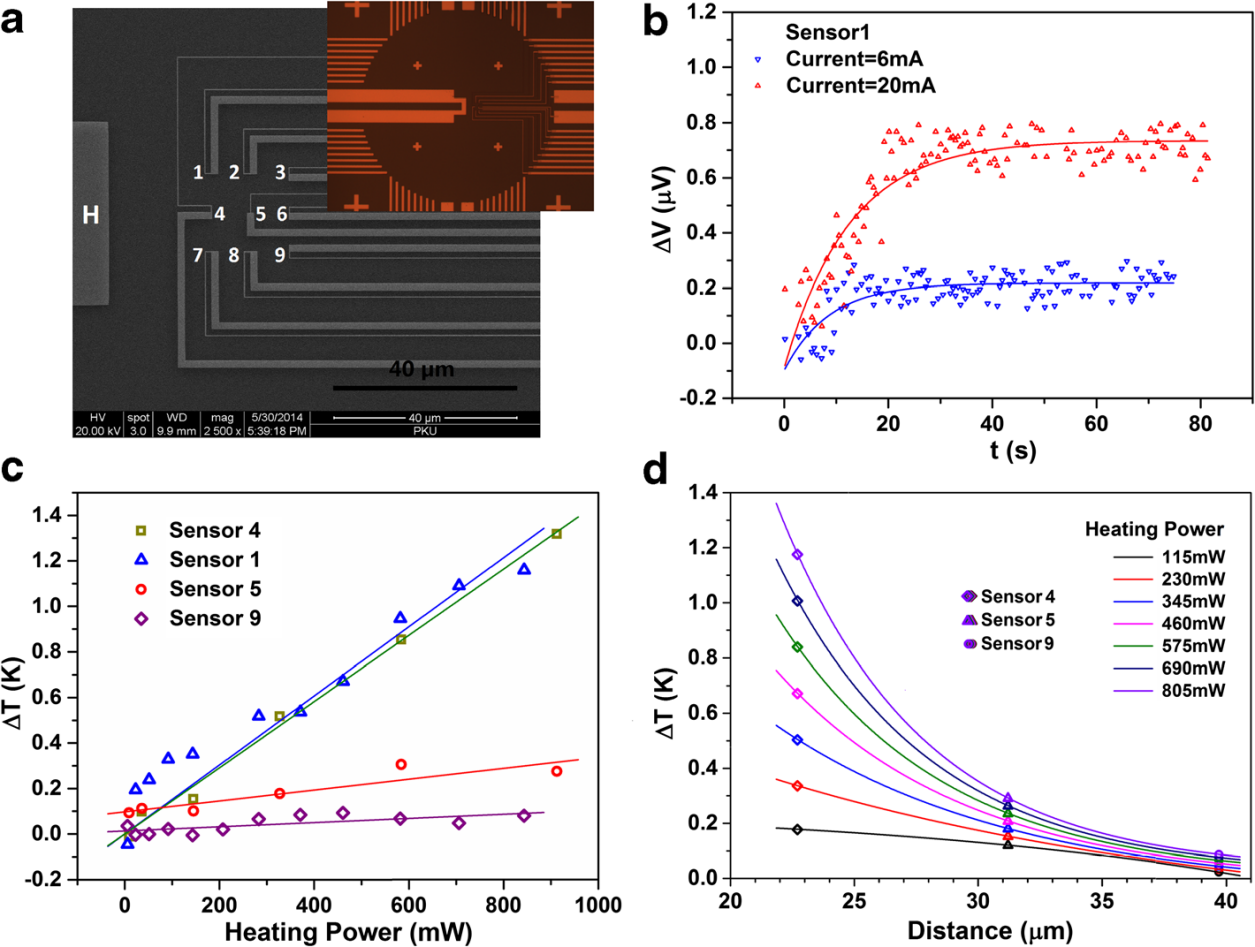
Experimental results of the sensor array. **a** SEM micrograph of a sensor array on a testing platform. The nine sensors are numbered from “1” to “9” with heater “H” located left aside. The heater is marked with “H”. The *inset* shows the microscopy graph of the central region of the device. **b** Raw output data of sensor 1 with heating currents of 6 and 20 mA. **c** Measurement results of sensors 1, 4, 5, and 9. Δ*T* values are calculated with Δ*S* of 0.75 μV/K and with Δ*V* being average value after stabilization. The data are linearly plotted. **d** Temperature decrease versus distance to the heater of sensors 4, 5, and 9 under seven different heating powers. The three sensors are 22.7, 31.2, and 39.7 μm away from the heater

Even with a distance of more than 20 μm away from the heater, the sensors work well in detecting a weak temperature increase *Δ*T. Figure 6 shows the experimental results measured from a platform device. Figure 6a is a SEM micrograph of a sensor array made on a testing platform device. The nine sensors are marked with numbers from “1” to “9”. The onsite heater is marked with “H”. The inset is a photograph for the central region of the device. For this sample, the open ends of the pre-patterned leads are about 200 μm away from the onsite heater.

Figure 6b plots the raw output voltage data of sensor 1 versus time, for two fixed heating current of 6 and 20 mA. The resistance of the heater is measured to be around 1450 Ω under a small excitation current. The output signals increase first and stabilize after around 20 and 40 s for the current of 6 mA and 20 mA, respectively, where we can see the stabilizing time increase with the current. After stabilization, the voltage has a fluctuation of about ±0.1 μV, corresponding to a temperature fluctuation of ±0.13 K. This is consistent with the physical process of heat transport along the SiO_2_/Si substrate surface. With further measurement in time, the output does not increase because an equilibrium has been established when the heating power and dissipation are balanced. Therefore, although signal fluctuation is observed in the steady state region, we take the average stabilized output voltage data for each fixed heating powers. In Figure 6c, the experimental data of four sensors, namely sensors 1, 4, 5, and 9 in Fig. 6a, are plotted. With an increasing heating power, sensors 1 and 4 detect the higher temperatures than those by sensors 5 and 9, as sensors 1 and 4 are closer to the heater. Clearly, for sensors 1 and 4, a small Δ*T* of 0.2–0.4 K is detectable, and the output signal responses linearly to the heating power, showing a good sensing performance. But for sensors 5 and 9, as they are too far away from the heating zone, they show a low signal to noise ratio.

Now we give a brief analysis for the correlation between detected Δ*T* and the distance to the heater for sensors 4, 5, and 9. The distance from the center of each sensor to the edge of the heater are 22.7, 31.2, and 39.7 μm, respectively. Figure 6d plots the Δ*T* value versus sensor distance under seven fixed heating powers from 115 to 805 mW. Here we assume the isothermal lines on the surface were the red curves shown in Additional file 1: Figure S1. As the distance increases, Sensors 3, 6 and 9 are expected to show similar results. The colorful diamonds, triangles, and circles represent the experimental data, and the solid curves are fitting results, which are only guidelines to eye for a qualitative analysis. It shows that Δ*T* values decrease dramatically with the distance. Δ*T* is almost negligible when the distance is over 40 μm. This result leads to a safe conclusion, that at the cold end region for the sensors where EBL-made cold ends overlap with pre-patterned leads, its 200-μm distance to the heating zone would make Δ*T* of the cold ends close to 0.

From the experimental data shown in Fig. 2a and Fig. 6c, one can see that our nano-submicron-stripe dual-beam Pd sensors work well in detecting local weak temperature changes on a solid surface. When the sensor-heater distance is as close as 2 μm, as shown in Fig. 2a, a local temperature fluctuation of 0.1 K is detectable. When the sensor heater distance is increased to 20 μm, the detectable Δ*T* is around 0.2–0.4 K. Therefore, we can expect that when the heating zone is located directly over the sensor, with an insulating space of 20–100 nm, for instance, the present dual-beam Pd sensors may ensure a resolution of 0.1 K with reliable stability.

## Conclusions

In this work, we reported the calibration and sensing performance of dozens of dual-beam temperature sensors made from 90-nm-thick Pd thin films. With a clear-cut shape, the total width of the sensors is squeezed to as small as 430 nm. These sub-micron sensors have stable and reproducible sensitivity of 0.7–1.2 μV/K, depending mainly on the width combination of each sansor. We also tested a combined fabrication procedure, where a central array of the Pd sensors was patterned with e-beam lithography and overlapped on patterned long micro-leads made with photolithography technique. The hybrid process remarkably reduced the device cost and showed the scaleup capability of the submicron sensors in a sophisticated device. The testing results showed that over an area of diameter 40–100 μm, the sensors could detect a weak surface temperature difference of 0.1–0.2 K. The merits of simple structure, stable performance, and compatible process to standard semiconductor techniques make the sub-micron sensors applicable in solid electronic devices, microfluidic systems, and even for bio-sensing at the cell level. For instance, it may detect local temperature distribution on an active electronic device with a better temperature resolution than what has been reported [33], or replace conventional thin-film thermocouples as the built-in sensor arrays in sophisticated lab-on-a-chip systems [34], and show better performance as they can also be fabricated with their sensing junctions open to the fluid channel [35].

## Additional file

Additional file 1: Figure S1. The red lines are the isothermal lines for the expected temperature distribution on the substrate surface. The sensors in the testing array are numbered the same series numbers from 1 to 9 as that in the main text (DOC 363 kb).
