# Supplementary material for: Performance of Nano-Submicron-Stripe Pd Thin-Film Temperature Sensors
Source: Nanoscale Res Lett. 2016 Jul 28;11:351. doi: 10.1186/s11671-016-1565-8 (PMC4963350; doi:10.1186/s11671-016-1565-8)
Supplement: Additional file 1: Figure S1. — The red lines are the isothermal lines for the expected temperature distribution on the substrate surface. The sensors in the testing array are numbered the same series numbers from 1 to 9 as that in the main text (DOC 363 kb). [file 11671_2016_1565_MOESM1_ESM.doc]

**Performance of nano-submicron-stripe Pd thin-film temperature sensors ― Supplementary information**

Xiaoye Huo, Jingjing Xu, Zhenhai Wang, Fan Yang, and Shengyong Xu*

We assume that the heat dissipation fronts on the surface, or the isothermal lines, were the red curves shown in Figure S1. As the distance increases, locations at Sensors 3, 6 and 9 are expected to show similar results, just as Sensor 1 and 4 show similar results in Figure 6c.


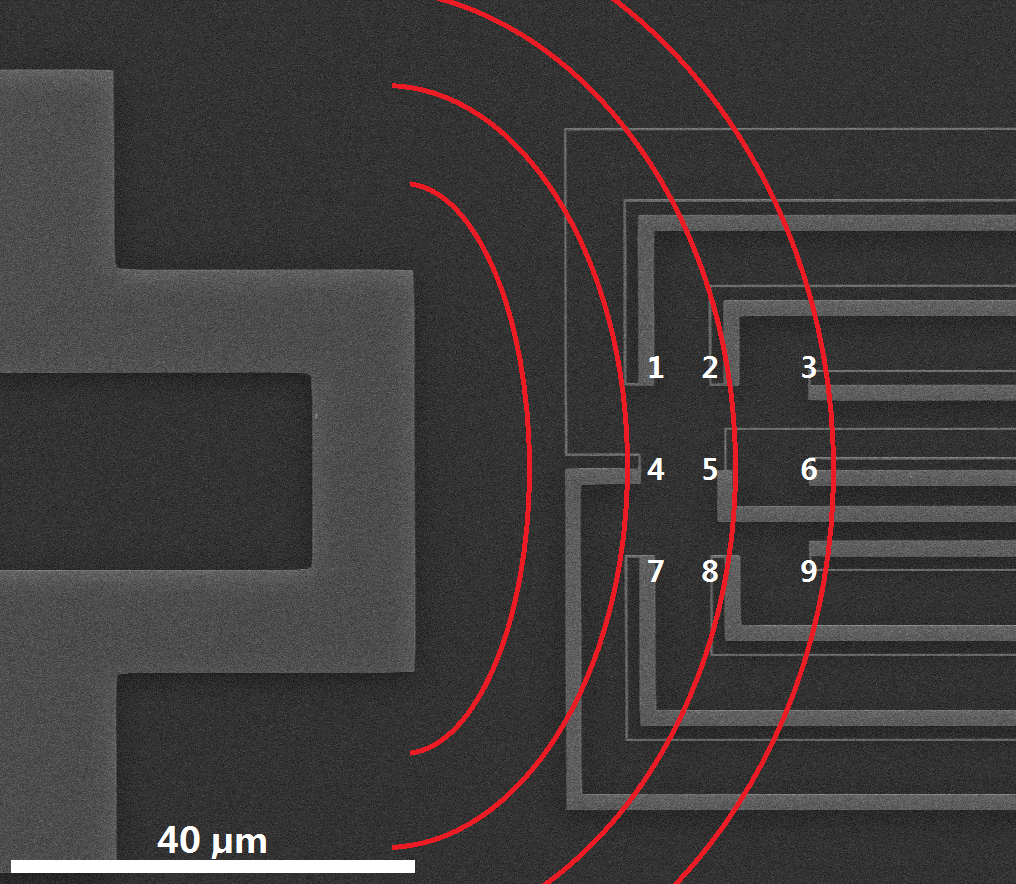


Figure S1 The red lines are the isothermal lines for the expected temperature distribution on the substrate surface. The sensors in the testing array are numbered the same series numbers from 1 to 9 as that in the main text.
